# Genetics and the heart rate response to exercise

**DOI:** 10.1007/s00018-019-03079-4

**Published:** 2019-03-27

**Authors:** Yordi J. van de Vegte, Balewgizie S. Tegegne, Niek Verweij, Harold Snieder, Pim van der Harst

**Affiliations:** 1 Department of Cardiology, University of Groningen, University Medical Center Groningen, Hanzeplein 1, 9700 RB Groningen, The Netherlands; 2 Department of Genetics, University of Groningen, University Medical Center Groningen, 9700 RB Groningen, The Netherlands; 3 Department of Epidemiology, University of Groningen, University Medical Center Groningen, 9700 RB Groningen, The Netherlands; 4 Durrer Center for Cardiogenetic Research, Netherlands Heart Institute, 3511 GC Utrecht, The Netherlands

**Keywords:** Heart rate increase, Heart rate recovery, Exercise, Genetics

## Abstract

The acute heart rate response to exercise, i.e., heart rate increase during and heart rate recovery after exercise, has often been associated with all-cause and cardiovascular mortality. The long-term response of heart rate to exercise results in favourable changes in chronotropic function, including decreased resting and submaximal heart rate as well as increased heart rate recovery. Both the acute and long-term heart rate response to exercise have been shown to be heritable. Advances in genetic analysis enable researchers to investigate this hereditary component to gain insights in possible molecular mechanisms underlying interindividual differences in the heart rate response to exercise. In this review, we comprehensively searched candidate gene, linkage, and genome-wide association studies that investigated the heart rate response to exercise. A total of ten genes were associated with the acute heart rate response to exercise in candidate gene studies. Only one gene (*CHRM2*), related to heart rate recovery, was replicated in recent genome-wide association studies (GWASs). Additional 17 candidate causal genes were identified for heart rate increase and 26 for heart rate recovery in these GWASs. Nine of these genes were associated with both acute increase and recovery of the heart rate during exercise. These genes can be broadly categorized into four categories: (1) development of the nervous system (*CCDC141*, *PAX2*, *SOX5,* and *CAV2*); (2) prolongation of neuronal life span (*SYT10*); (3) cardiac development (*RNF220* and *MCTP2*); (4) cardiac rhythm (*SCN10A* and *RGS6*). Additional 10 genes were linked to long-term modification of the heart rate response to exercise, nine with heart rate increase and one with heart rate recovery. Follow-up will be essential to get functional insights in how candidate causal genes affect the heart rate response to exercise. Future work will be required to translate these findings to preventive and therapeutic applications.

**Electronic supplementary material:**

The online version of this article (10.1007/s00018-019-03079-4) contains supplementary material, which is available to authorized users.

## Introduction

The regulation of resting heart rate is complex; autonomic tone, central and peripheral reflexes, hormonal influences, and factors intrinsic to the heart are all important determinants [1, 2]. Despite recent developments in the understanding of the complex interplay of the plethora of biological mechanisms influencing resting heart rate [3], our understanding is still incomplete.

The acute heart rate response to exercise, heart rate increase during and heart rate recovery after exercise, offers unique insights into cardiac physiology compared to heart rate in rest and can therefore be exploited to obtain additional information on cardiac function [4]. Impaired increase of heart rate during exercise (chronotropic incompetence) and an attenuated heart rate recovery have been associated with all-cause mortality and sudden cardiac death in healthy individuals [5–7] and in those with cardiac disease, including individuals with heart failure [8] and coronary artery disease [9]. Regular endurance exercise training has been proven to shift the cardiac autonomic balance towards vagal dominance [10]. The long-term response of heart rate to exercise results in favourable changes in chronotropic function, including decreased resting and submaximal heart rate as well as increased heart rate recovery [11].

Both the acute and long-term responses of heart rate to exercise have been shown to have a large heritable component [12–17]. Development in the understanding of the human genome and genetic analysis enables researchers to investigate the possible molecular mechanisms underlying interindividual differences in the acute and long-term heart rate response to exercise [18]. In this review, we summarize the current knowledge of the acute and long-term heart rate response to exercise, with a focus on the genetic contribution. In addition, we identify gaps in our knowledge and discuss possible future directions that might be of interest to enhance the understanding of the heart rate response to exercise and consider its potential clinical applications.

## Acute response

### Heart rate increase

In general, the regulation of the circulatory system during exercise involves several adaptations. These adaptations include dilatation of resistance vessels in the active muscles, a decrease in vagal outflow to the heart, followed by an increase of sympathetic outflow. If exercise is intense, the cholinergic fibers to the adrenal medulla are also activated, resulting in release of epinephrine into the circulation [19]. Under normal physiological conditions, this results in increased venous return, contractility, and heart rate [20]. In turn, ejection fraction increases due to a greater ejection of blood at the end of systole and increased diastolic filling of the ventricles as the duration of the systole decreases with increased heart rate [20].

The increase of heart rate during exercise is for a major part attributable to the decrease in vagal tone followed by an increase in sympathetic outflow and an increase in levels of circulating catecholamines [19]. It has been shown that a substantial component of interindividual differences in the heart rate increase during exercise is genetically determined, with heritability estimates ranging from 0.17 to 0.32 (Table 1) [12, 14, 15]. This suggests that genetic analyses may identify novel biological mechanisms involved in the regulation of heart rate response to exercise.

**Table 1:** Heritability estimates for the acute and long-term effect of exercise on heart rate response

Heritability type	Heritability	Type of exercise	Population	*N*	Author, year
Acute response: heart rate increase					
Family	0.32	Submaximal treadmill test	General population	2053	Ingelsson et al. (2007) [12]
SNP-based	0.22	Submaximal bicycle	General population	58,818	Verweij et al. (2018) [14]
SNP-based	0.17	Submaximal bicycle	General population	66,800	Ramirez J et al. (2018) [15]
Acute response: heart rate recovery					
Family^a^	0.34	Submaximal treadmill test	General population	2053	Ingelsson et al. (2007) [12]
Twins and sibling^b^	0.60 and 0.65	Maximal bicycle	General population	491	Nederend et al. (2016) [13]
SNP-based^c^	0.22	Submaximal bicycle	General population	58,818	Verweij et al. (2018) [14]
SNP-based^c^	0.12	Submaximal bicycle	General population	66,665	Ramirez et al. (2018) [15]
Trainings response: heart rate increase					
Family^d^	0.34	Submaximal bicycle	General population	481	An et al. (2003) [17]
Family^d^	0.36	Submaximal bicycle	Participants with high blood pressure	529	Rice et al. (2002) [16]

Heritability estimates for the acute and long-term effect of exercise on heart rate response

^a^Heart rate recovery measured after 180 s

^b^Heart rate recovery measured after, respectively, 60 and 180 s

^c^Heart rate recovery measured after 60 s

^d^20 weeks during endurance training program at submaximal (50W) levels

**Fig. 1:**
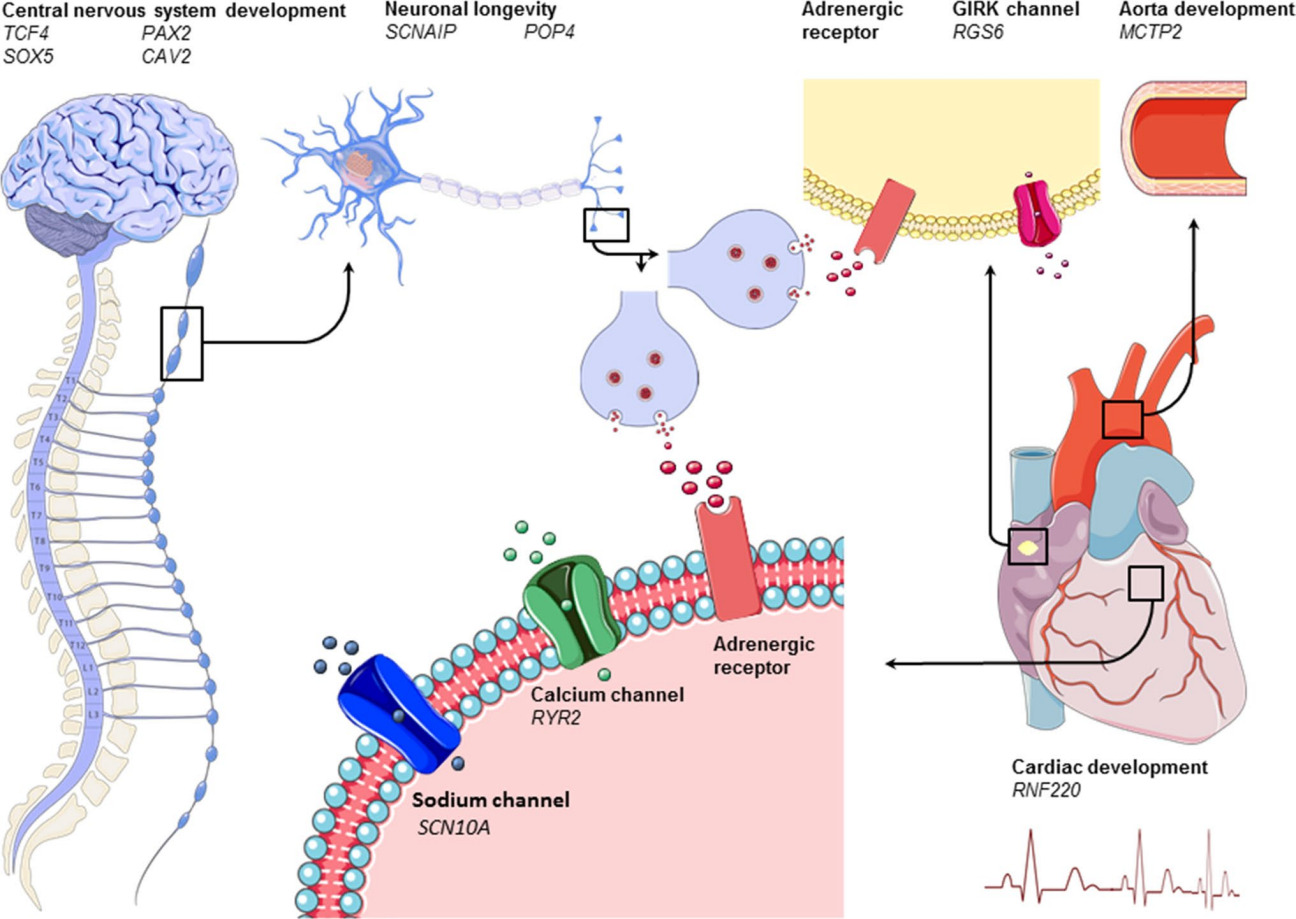
Graphical representation of genes (shown in italic) involved in acute heart rate increase during exercise grouped by working mechanism (shown in bold). The left and left upper part of the figure shows the nervous system. The middle upper part zooms in on a peripheral sympathetic neuron and its synapse. The heart is displayed on the right; the upper right of the figure shows the aorta with next to it a pacemaker cell in the cardiac sinus node. In the middle of the figure, below, we zoom in on cardiac tissue and receptors. Adrenergic receptors are shown in red. Sodium, potassium, and calcium channels are shown in red, pink, and green, respectively

Several studies have focussed on identifying genetic determinants that explain interindividual differences in heart rate increase during exercise. Genes investigated in these studies are summarized in Table 2, shown in Fig. 1, and are further discussed here. The *ACE* gene was one of the first candidate genes thoroughly investigated for its possible relationship with the heart rate response to exercise [21]. Genetic association studies focusing on the effect of the *ACE* gene on heart rate increase during exercise reported many conflicting results [12, 22–25]. Some studies tested genes for their indirect effect on the sympathetic nervous system. One study observed that the *NOS3* gene, which produces nitric oxide, was associated with heart rate increase during exercise [26]. Although nitric oxide is mostly known for its vasodilatory effects, it is also thought to have a modulating effect on the parasympathetic and sympathetic nervous system [27]. *GNAS1* was found to be associated with heart rate increase during exercise as well [28]. This gene encodes the G protein α-subunit that influences the sympathetic nervous system as it enables the coupling between adenylyl cyclase and β1-adrenergic receptors. On the other hand, many studies brought forward genes based on their direct involvement in the sympathetic nervous system, and associations were found with the *ADRB1* [29] and *ADRB2* [30] genes, which both encode for β-adrenergic receptors. Interestingly, many previous findings could not be replicated in the Framingham Offspring study, which investigated multiple genes instead of focusing on a single gene. In this study, associations were found with the *ADRA1A* and *ADRA1D* [12]. These genes encode for α-adrenergic receptors that are mainly involved in smooth muscle cell contraction during sympathetic stimulation [12]. However, associations with the *ADRB1* and *ADRB2* genes could not be re-established [12].

**Table 2:** Summary of genes involved in acute heart rate increase

Gene	Variant	Chromosome/position	Minor/major allele/MAF	Type of study	Increase/decrease	*P* value	Type of exercise test	Population	Author; year
*ACE* ^a^	Del intron 16	17:63488529	Deletion/insertion	Candidate	–	> 5.00 × 10^−2^	Maximal and submaximal bicycle	General	Rankinen et al. (2000) [25]
*ADRA1A*	rs544215	8:26712028	C/T/0.46	Candidate	↓	5.00 × 10^−3^	Standard Bruce	General	Ingelsson et al. (2007) [12]
*ADRA1D*	rs3787441	20:4205059	G/A/0.27	Candidate	↓	7.00 × 10^−3^	Standard Bruce	General	Ingelsson et al. (2007) [12]
*ADRB1*	rs1801253	10:114045297	C/G/0.28	Candidate	↓	< 5.00 × 10^−2^	Maximal	Patients in cardiac rehab	Defoor et al. (2005) [29]
*ADRB1*	rs1801252	10:114044277	G/A/0.21	Candidate	↓	< 5.00 × 10^−2^	Maximal	Patients in cardiac rehab	Defoor et al. (2005) [29]
*ADRB2*	rs1042713	5:148826877	A/G/-	Candidate	↓	< 5.00 × 10^−2^	Hand grip test	General	Eisenach et al.(2003)[30]
*CAV2*	rs28495552	7:116113744	C/G/0.50	GWAS	↓	2.80 × 10^−11^	Submaximal bicycle	General	Ramirez et al. (2018) [15]
*CCDC141*	rs10497529	2:179839888	A/G/0.04	GWAS	↑	2.50 × 10^−9^	Submaximal bicycle	General	Ramirez et al. (2018) [15]
*GNAS1* ^b^	rs7121	20:58903752	C/T/0.37	Candidate	↑	<5.00 × 10^−2^	Ergometer	Referred for exercise test	Nieminen et al. (2006) [28]
*HMGA2*	rs1480470	12:66412130	A/G/0.37	GWAS	↑	3.40 × 10^−08^	Submaximal bicycle	General	Ramirez et al. (2018) [15]
*MCTP2*	rs12906962	15:95312071	C/T/0.32	GWAS	↓	3.50 × 10^−13^	Submaximal bicycle	General	Ramirez et al. (2018) [15]
*MCTP2*	rs12906962	15:95312071	C/T/0.33	GWAS	↓	2.70 × 10^−14^	Submaximal bicycle	General	Verweij et al. (2018) [14]
*NOLA* ^c,d^	rs6847149	4:111157701	–	GWAS	–	2.74 × 10^−06^	Standard Bruce	General	Vasan et al. (2007) [34]
*NOS3* ^e^	rs1799983	7:150999023	T/G/0.26	Candidate	↓	4.00 × 10^−2^	Naughton protocol	Post-menopausal women	Hand et al. (2006) [26]
*PAX2*	rs11190709	10:102552663	G/A/0.12	GWAS	↑	1.30 × 10^−11^	Submaximal bicycle	General	Ramirez et al. (2018) [15]
*POP4*	rs12986417	19:30109533	A/G/0.35	GWAS	↓	1.00 × 10^−9^	Submaximal bicycle	General	Verweij et al. (2018) [14]
*POP4*	rs7255293	19:30104198	G/A/0.42	GWAS	↓	3.20 × 10^−9^	Submaximal bicycle	General	Ramirez et al.(2018)[15]
*PPIL1*	rs236352	6:36817113	A/G/0.34	GWAS	↑	6.40 × 10^−10^	Submaximal bicycle	General	Ramirez et al. (2018) [15]
*RGS6*	rs17180489	14:72885471	C/G/0.14	GWAS	↑	2.50 × 10^−11^	Submaximal bicycle	General	Verweij et al. (2018) [14]
*RNF220*	rs272564	1:45012273	C/A/0.28	GWAS	↓	7.40 × 10^−12^	Submaximal bicycle	General	Ramirez et al. (2018) [15]
*RP1L1*	rs58065122	8:10526186	A/G/0.42	GWAS	↑	3.90 × 10^−10^	Submaximal bicycle	General	Ramirez et al. (2018) [15]
*RYR2* ^c,d^	rs2819770	1:234237045	–	GWAS	–	3.53 × 10^−6^	Standard Bruce	General	Vasan et al. (2007) [34]
*SCN10A*	rs7433723	3:38784957	G/A/0.42	GWAS	↓	4.50 × 10^−8^	Submaximal bicycle	General	Ramirez et al. (2018) [15]
*SNCAIP*	rs4836027	5:121866990	C/T/0.32	GWAS	↓	1.70 × 10^−15^	Submaximal bicycle	General	Verweij et al. (2018) [14]
*SNCA1P*	rs4836027	5:121866990	C/T/0.31	GWAS	↓	9.90 × 10^−21^	Submaximal bicycle	General	Ramirez et al. (2018) [15]
*SOX5*	rs4246224	12:24784139	A/G/0.15	GWAS	↑	1.80 × 10^−14^	Submaximal bicycle	General	Ramirez et al. (2018) [15]
*SYT10*	rs1343676	12:33537387	T/C/0.51	GWAS	↓	1.50 × 10^−11^	Submaximal bicycle	General	Ramirez et al. (2018) [15]
*TCF4*	rs1125313	18:52859261	C/A/0.50	GWAS	↑	3.90 × 10^−9^	Submaximal bicycle	General	Ramirez et al. (2018) [15]

Genes found to be associated with heart rate increase during exercise are shown in alphabetical order and are then ordered on the year published. Variation stands for either an SNP or deletion/insertion mutation. MAF stands for Minor Allele Frequency. Effects of a variant (in- or decrease) on heart rate increase during exercise are shown for the Minor Allele. Candidate stands for candidate gene study. GWAS stands for genome-wide association study. A hyphen is shown in case information which was not reported

^a^Results from only one candidate gene study on ACE are shown; largest study was chosen; G allele in case of deletion; in case of insertion ATACAGTCACTTTTTTTTTTTTTTTGAGACGGAGTCTCGCTCTGTCGCCC

^b^Statistics from gene time of exercise interaction are showed

^c^Standard Bruce protocol is a maximal exercise treadmill test

^d^Statistics from the generalized estimating equations (GEE) tests are shown; Alleles were not mentioned in this article. None reached genome-wide significance; however, these were the most suggestive results

^e^Naughton protocol is a maximal exercise treadmill test

Although these studies were important for laying the foundation of our knowledge on the genetic determinants of heart rate increase during exercise, they failed to yield a comprehensive view by focusing on one or only a few genes. The Framingham Offspring study was the first to address these issues by conducting an early genetic linkage analysis on heart rate increase and recovery. However, not one genetic signal reached the appropriate significance level, which can possibly be attributed to the relatively low sample size of this study (*n * = 2982) [12]. In addition, linkage analyses have been shown to be less successful when applied to polygenic traits such as heart rate response to exercise [31], in part because of their limited power to detect the effect of common alleles with modest effects on disease [32].

More recently, genome-wide association studies (GWASs) were introduced. GWASs do have the potential to detect common alleles with modest effects on disease, since this method allows an unbiased and comprehensive search across the genome for single nucleotide polymorphisms (SNPs) [33]. The first GWAS on heart rate increase during exercise found *GAR1* and *RYR2* genes to be associated [34]. *GAR1* is required for ribosome biogenesis and telomere maintenance. However, its specific function and how it possibly interacts with heart rate increase during exercise is unknown. *RYR2* encodes a calcium channel that mediates calcium release from the sarcoplasmic reticulum into the cytoplasm and is therefore essential in triggering cardiac muscle contraction (Table 2, Fig. 1). *RYR2* mutations in humans are associated with arrhythmogenic right-ventricular dysplasia and catecholaminergic polymorphic ventricular tachycardia. Interestingly, although caused by a different mutation in the *RYR2* gene, both diseases are known to cause exercise-induced tachycardia [35–37]. However, these associations did not reach genome-wide significance, which might be due to the low sample size (*n* = 1238) [34].

Increasing the sample size for GWASs has been simplified by the development of inexpensive SNP arrays. Two GWASs were recently conducted on the acute heart rate response to exercise in the same cohort of the UK Biobank [14, 15]. The discussion of methodological differences between these studies has been published previously [38] and is beyond the scope of the current review. However, a summary of important differences is necessary to understand different genes found between the two studies. One difference is that the first study by Verweij et al. had a slightly lower sample size, since they used only echocardiography (ECG) measurements and did not include heart rate measurements derived by the UK Biobank itself. Another difference is that the study of Verweij et al. applied a more stringent threshold to claim a genome-wide significant level to be true (strategy to reduce the risk of type-1 errors) compared to the study published later by Ramirez et al. (*p* < 8.3 × 10^−9^ vs *p* < 5.0 × 10^−8^, respectively).

Of special interest are three genes that were found to be associated with heart rate increase during exercise in both studies, which are *SNCAIP, MCTP2,* and *POP4* [14, 15]. The exact mechanism of *SCNAIP* is not known so far; however, studies in mice have shown that *SCNAIP* plays a role in neuronal degeneration (Table 2, Fig. 1) [39, 40]. *POP4* is involved in the processing of precursor RNAs [41] and in the DNA damage response [42], thus preventing accumulation of deleterious mutations and DNA lesions and therefore potentially preventing genomic instabilities and carcinogenesis and prolonging neuronal life span. The *MCTP2* gene is more specific to cardiac tissue. A mutation in the *MCTP2* is known to cause left-ventricular outflow tract malformations in humans, which may alter the pressure within the ventricular outflow tract. Baroreceptors are densely located in this region and altered blood pressure could therefore lead to altered autonomic feedback on heart rate (Table 2, Fig. 1) [43]. Several other candidate genes found in these studies already provide a biological hypothesis to account for the associations with heart rate response to exercise. These genes can be broadly categorized into four categories, that is: (1) development of the nervous system, including the *CCDC141* [44, 45], *TCF4* [46, 47]*, PAX2* [48]*, SOX5* [49, 50], and *CAV2* [51] genes; (2) prolongation of neuronal life span, including the *SYT10* [52] gene; (3) cardiac development and disease, including RNF220 [53, 54] gene; and, finally, (4) genes involved in cardiac rhythm, including *SCN10A* [55] and *RGS6* [56, 57]. Of these, *CCDC141, CAV2*, *SYT10, RNF220,* and *SCN10A* were more strongly associated with heart rate recovery after exercise (Tables 2, 3) and will be therefore discussed later. *TCF4* is involved in the initiation of neuronal differentiation. Clinically, a mutation in *TCF4* is known to cause Pitt-Hopkins syndrome, a severe congenital encephalopathy characterized by intellectual disability, developmental problems, seizures, breathing problems, and typical facial features [46, 47]. *PAX2* encodes paired box gene 2 and is important in the early embryonic development as well. It is mostly known for its involvement in development of the kidney and urinary tract, since it is linked to papillorenal syndrome [58] and focal segmental glomerulosclerosis [59]. However, downstream target effectors of *PAX2* have been hypothesized to be involved in neuronal development because of their supposed effect on the CHARGE syndrome [48]. *SOX5* is involved in the regulation of chondrogenesis and the development of the nervous system [50]. In mice, it was found that loss of *SOX5* resulted in decreased neuronal differentiation and secondary migrational abnormalities [49]. Mutations of the *SOX5* gene in humans are known to cause the Lamb–Shaffer syndrome, which is characterized by speech delay, behavioural problems, and nonspecific dysmorphic features [50]. *RGS6* is part of the regulation mechanism of the parasympathetic nervous system in the heart [56, 57]. It decreases muscarinic type 2 receptor (M2R) signalling in the sinoatrial node by rapidly terminating Gβγ signalling [56, 57]. In mice, it was shown that *RGS6* knockdown removes the negative regulation of Gβγ leading to enhanced G protein-coupled inwardly rectifying potassium channel (GIRK)-induced sinoatrial and atrioventricular node hyperpolarization [56, 57]. It was therefore concluded that normal function of *RGS6* is important for preventing parasympathetic override and severe bradycardia [56]. Its involvement in the parasympathetic nervous system was recently established in another GWAS in which it was found to be associated with heart rate variability [60], which is known to reflect parasympathetic activity [61]. Concerning heart rate increase during exercise, normal function of *RGS6* probably facilitates parasympathetic withdrawal leading to the possibility to increase heart rate (Fig. 1).

**Table 3:** Summary of genes involved in acute heart rate recovery

Gene	Variant	Chromosome/position	Minor/major allele/MAF	Increase/decrease	*P* value	Type of study	Type of exercise test	Time (seconds after exercise)	Population	Author; year
ACE	–	–	Deletion/insertion	–	< 1.00 × 10^−2^	Candidate	Running for 25 min with heart rate between 165 and 170	1800	Athletes	Voroshin et al. (2008) [22]
ACHE	rs3757868	7:100482720	A/G/0.18	↓	5.60 × 10^−24^	GWAS	Submaximal bicycle	40	General	Verweij et al. (2018) [14]
ACHE	rs3757868	7:100482720	A/G/0.18	↓	6.90 × 10^−11^	GWAS	Submaximal bicycle	50	General	Ramirez et al. (2018) [15]
ADRA1B^a^	rs11953285	5:159324389	C/A/0.13	↑	1.00 × 10^−2^	Candidate	Standard Bruce^b^	180	General	Ingelsson et al. (2007) [12]
ADRA2B^c^	del301–303	2:96115249	./AAGAGGAG/0.37	↓	1.00 × 10^−2^	Candidate	Maximal bicycle	60	General	Kohli et al. (2015) [65]
ALG10B	rs4533105	12:38214611	C/T/0.43	↓	1.90 × 10^−13^	GWAS	Submaximal bicycle	50	General	Ramirez et al. (2018) [15]
BCAT1	rs4963772	12:24758480	A/G/0.15	↑	1.20 × 10^−28^	GWAS	Submaximal bicycle	40	General	Verweij et al. (2018) [14]
BCL11A	rs1372876	2:60025963	A/C/0.41	↓	3.30 × 10^−9^	GWAS	Submaximal bicycle	50	General	Ramirez et al. (2018) [15]
CAV2	rs1997571	7:116198621	G/A/0.48	↓	1.70 × 10^−12^	GWAS	Submaximal bicycle	20	General	Verweij et al. (2018) [14]
CAV2	rs2109514	7:116159961	A/G/0.50	↑	7.10 × 10^−10^	GWAS	Submaximal bicycle	50	General	Ramirez et al. (2018) [15]
CCDC141,TTN	rs17362588	2:179721046	A/G/0.08	↓	3.10 × 10^−9^	GWAS	Submaximal bicycle	10	General	Verweij et al. (2018) [14]
CCDC141,TTN	rs35596070	2:179759692	A/C/0.14	↓	4.20 × 10^−13^	GWAS	Submaximal bicycle	10	General	Verweij et al. (2018) [14]
CHRM2	rs324640	7:136146251	C/T/0.39	↓	8.00 × 10^−3^	Candidate	Maximal bicycle	60	General	Hautala (2006) [66]
CHRM2	rs8191992	7:136158563	A/T/0.37	↓	2.50 × 10^−3^	Candidate	Maximal bicycle	60	General	Hautala (2006) [66]
CHRM2^d^	rs324640	7:136146251	C/T/0.44	↓	1.70 × 10^−3^	Candidate	Symptom-limited maximal bicycle	60	Post-MI	Hautala (2009) [67]
CHRM2^d^	rs8191992	7:136158563	A/T/0.43	↓	1.60 × 10^−3^	Candidate	Symptom-limited maximal bicycle	60	Post-MI	Hautala (2009) [67]
CHRM2	rs17168815	7:136624621	T/G/0.16	↓	1.10 × 10^−14^	GWAS	Submaximal bicycle	50	General	Verweij et al. (2018) [14]
CHRM2	rs6943656	7:136639436	G/A/0.16	↓	2.30 × 10^−10^	GWAS	Submaximal bicycle	50	General	Ramirez et al. (2018) [15]
CLPB, INPPL1	rs7130652	11:71984398	T/G/0.07	↑	3.40 × 10^−11^	GWAS	Submaximal bicycle	10	General	Verweij et al. (2018) [14]
CNTN3	rs34310778	3:74783408	C/T/0.43	↑	1.00 × 10^−9^	GWAS	Submaximal bicycle	30	General	Verweij et al. (2018) [14]
CNTN3	rs6549649	3:74786491	C/G/0.64	↑	1.40 × 10^−9^	GWAS	Submaximal bicycle	50	General	Ramirez et al. (2018) [15]
GNG11	rs180238	7:93550447	C/T/0.35	↓	2.20 × 10^−12^	GWAS	Submaximal bicycle	40	General	Verweij et al. (2018) [14]
GRIK2	rs2224202	6:102053814	A/G/0.19	↑	5.80 × 10^−9^	GWAS	Submaximal bicycle	20	General	Verweij et al. (2018) [14]
KCNH8	rs73043051	3:18883863	C/T/0.22	↑	7.80 × 10^−9^	GWAS	Submaximal bicycle	50	General	Verweij et al. (2018) [14]
MCTP2	rs12906962	15:95312071	C/T/0.32	↓	5.10 × 10^−9^	GWAS	Submaximal bicycle	50	General	Ramirez et al. (2018) [15]
MED13L	rs61928421	12:116227249	T/C/0.07	↓	4.30 × 10^−15^	GWAS	Submaximal bicycle	40	General	Verweij et al. (2018) [14]
MED13L	rs11067773	12:11622895	C/T/0.09	↓	3.10 × 10^−11^	GWAS	Submaximal bicycle	50	General	Ramirez et al. (2018) [15]
NDUFA11	rs12974440	19:5894386	A/G/0.08	↓	2.40 × 10^−10^	GWAS	Submaximal bicycle	10	General	Verweij et al. (2018) [14]
NDUFA11	rs12974991	19:5894584	A/G/0.09	↓	2.10 × 10^−9^	GWAS	Submaximal bicycle	50	General	Ramirez et al. (2018) [15]
NEGR1	rs61765646	1:72723211	A/T/0.19	↑	1.10 × 10^−13^	GWAS	Submaximal bicycle	10	General	Verweij et al. (2018) [14]
PAX2	rs4917911	10:102559421	G/A/0.11	↑	6.60 × 10^−15^	GWAS	Submaximal bicycle	50	General	Ramirez et al. (2018) [15]
PAX2	rs7072737	10:102552663	G/A/0.11	↑	1.10 × 10^−17^	GWAS	Submaximal bicycle	40	General	Verweij et al. (2018) [14]
PRDM6	rs151283	5:122446619	A/C/0.28	↓	1.60 × 10^−10^	GWAS	Submaximal bicycle	50	General	Verweij et al. (2018) [14]
PRKAG2^e^	rs1029947	7:150713400	–	–	9.20 × 10^−7^	GWAS	Standard Bruce^b^	180	General	Vasan et al. (2007) [34]
PRKAG2^e^	rs1029946	7:150713454	–	–	3.89 × 10^−6^	GWAS	Standard Bruce^b^	180	General	Vasan et al. (2007) [34]
RGS6	rs150330648	14:72844765	T/G/0.01	↓	4.30 × 10^−8^	GWAS	Submaximal bicycle	50	General	Ramirez et al. (2018) [15]
RNF220	rs272564	1:45012273	C/A/0.29	↓	1.40 × 10^−12^	GWAS	Submaximal bicycle	50	General	Verweij et al. (2018) [14]
RNF220	rs272564	1:45012273	C/A/0.28	↓	8.80 × 10^−10^	GWAS	Submaximal bicycle	50	General	Ramirez et al. (2018) [15]
SCN10A	rs6795970	3:38766675	A/G/0.40	↓	2.60 × 10^−8^	GWAS	Submaximal bicycle	50	General	Ramirez et al. (2018) [15]
SERINC2	rs11589125	1:31894396	T/C/0.06	↑	6.60 × 10^−9^	GWAS	Submaximal bicycle	50	General	Verweij et al. (2018) [14]
SKAP	rs2158712	7:26582733	T/A/0.48	↓	2.80 × 10^−13^	GWAS	Submaximal bicycle	10	General	Verweij et al. (2018) [14]
SNCAIP	rs1993875	5:121869310	C/G/0.30	↓	9.50 × 10^−9^	GWAS	Submaximal bicycle	50	General	Ramirez et al. (2018) [15]
SOX5	rs112630705	12:24773919	A/G/0.15	↑	3.20 × 10^−11^	GWAS	Submaximal bicycle	50	General	Ramirez et al. (2018) [15]
SYT10	rs6488162	12:33593127	T/C/0.42	↓	2.60 × 10^−66^	GWAS	Submaximal bicycle	10	General	Verweij et al. (2018) [14]
SYT10	rs2218650	12: 33734783	A/G/0.15	↓	1.10 × 10^−26^	GWAS	Submaximal bicycle	50	General	Ramirez et al. (2018) [15]

Genes found to be associated with heart rate recovery after exercise are shown in alphabetical order and are then ordered on the year published. Mutation stands for either a SNP or deletion/insertion mutation. MAF stands for Minor Allele Frequency. Effect of variant (increase or decrease) on heart rate increase during exercise is shown for the minor allele. Candidate stands for candidate gene study. GWAS stands for genome-wide association study. MI stands for myocardial infarction. A hyphen is shown in case information which was not reported

^a^Insignificant after correction for multiple testing

^b^Standard Bruce protocol was used

^c^Heart rate recovery constant was used as measurement. This fits heart rate recovery to a first-order exponential decay curve

^d^Compared lowest and highest HRR quartile. Linear model showed no significant association

^e^Statistics from the family-based association tests are shown; Alleles were not mentioned in this article. None reached genome-wide significance. Results shown are the strongest associations

Interestingly, none of the genes investigated in candidate gene studies were found to be associated with heart rate increase in any of the three GWASs. This is in line with the previous work in which early candidate gene studies were difficult to replicate [62, 63]. Two genes, *HMGA2* and *PPIL1*, shown in Table 2 have not been discussed so far. *PPIL1* is a gene that was recently found to be associated with heart rate variability as well [60]. However, to our knowledge, there is no current biological hypothesis to explain the association between *PPIL1* or *HMGA2* and heart rate increase during exercise.

### Heart rate recovery

**Fig. 2:**
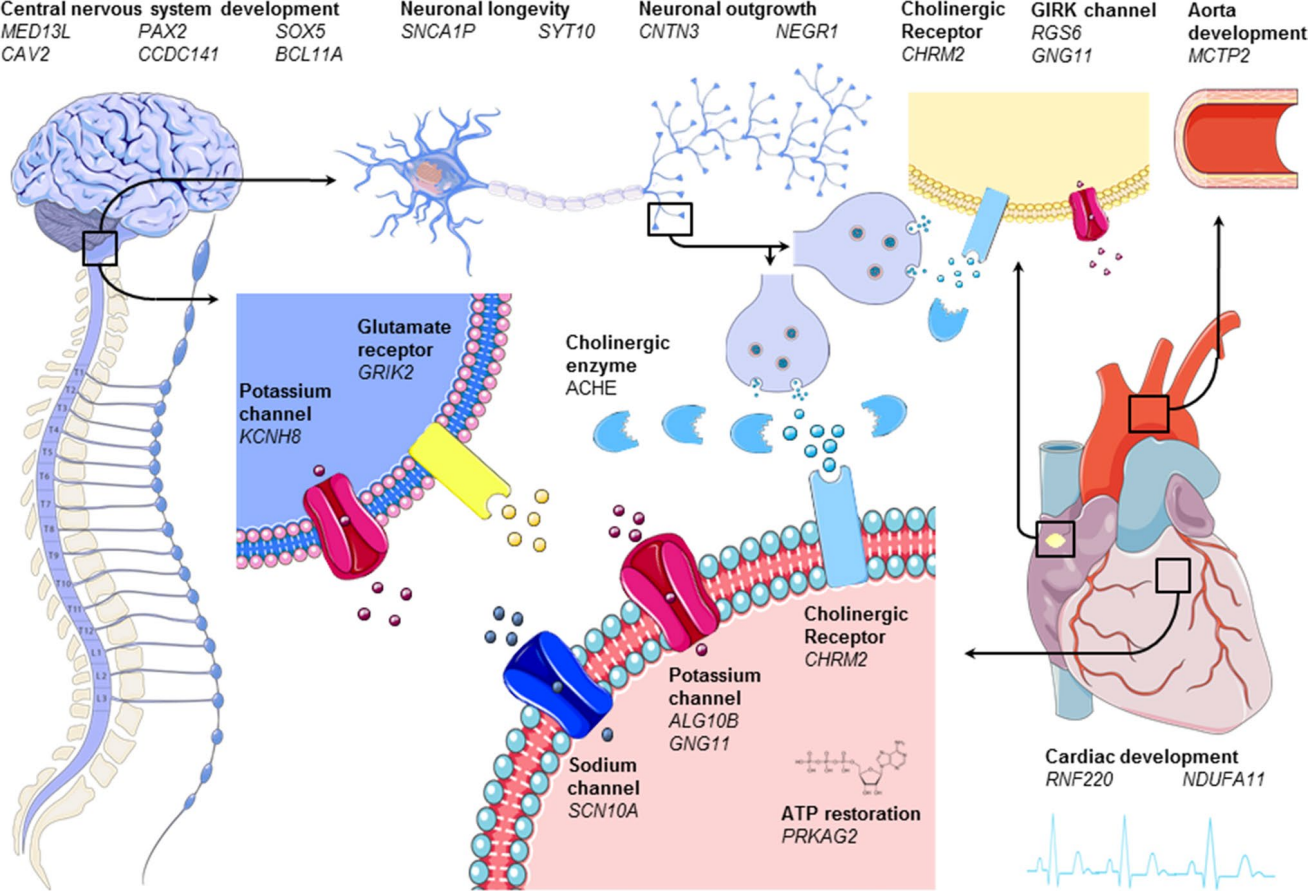
Graphical representation of genes (shown in italic) involved in acute heart rate recovery after exercise grouped by working mechanism (shown in bold). The left and left upper part of the figure shows the nervous system. The middle upper part zooms in on a parasympathetic neuron of the vagus nerve (twice) and its synapse. Note that although we zoom in on the brain stem (which is the main location of parasympathetic nuclei that innervate the vagus nerve), we actually show a peripheral parasympathetic neuron of the vagus nerve. The heart is displayed on the right; the upper right of the figure shows the aorta with next to it a pacemaker cell in the cardiac sinus node. In the middle of the figure, below, we zoom in on cardiac tissue and receptors. Cholinergic receptors and enzymes are shown in light blue and glutamate receptors in yellow. Sodium and potassium channels are shown in red and pink, respectively

Heart rate recovery is characterized by increased parasympathetic tone followed by sympathetic withdrawal, which follows an inversed gradient pattern compared to heart rate increase [19]. It was elegantly shown in a dual-blockade study that especially parasympathetic reactivation is essential for interindividual differences in heart rate recovery [64]. However, the exact mechanisms underlying these differences remain to be determined. Twin, family, and GWA studies estimated the genetic component to interindividual differences of heart rate recovery after one minute to range between 0.12 and 0.60 (Table 1). Therefore, genetic studies may yield novel insights into heart rate recovery. All genetic determinants investigated for their potential causal role in interindividual differences in heart rate recovery are summarized in Table 3 and are discussed below. An illustration of possible causal genes and how they are supposed to influence acute heart rate recovery after exercise is shown in Fig. 2.

Initially, the same candidate genes were proposed for heart rate recovery as for heart rate increase. For example, the *ACE* gene was found to be related to heart rate recovery in one candidate gene study as well [22]. Another study found *ADRA1B* and *ADRA2B* to be associated with heart rate recovery (Table 3) [12]. The association between *ADRA2B* gene and heart rate recovery was also found in another candidate gene study [65]. Other studies focused primarily on the parasympathetic nervous system represented by the *CHRM2* gene. The minor alleles of the rs324640 and rs8191992 SNPs found in *CHRM2* region were found to be associated with a lower heart rate recovery in the general population [66] and in patients with a history of myocardial infarction [67]. In addition, these minor alleles increased chances of death to coronary artery disease in the latter group [67].

The problem of biased selection of candidate genes has been solved by conducting GWASs as previously stated. The first GWAS on the acute heart rate response to exercise found heart rate recovery measured 3 min post-exercise to be associated with *PRKAG2,* though this association did not reach genome-wide significance. *PRKAG2* is involved in the regulation of ATP restoration after periods of ATP depletion and therefore might influence the return of heart rate to its initial state (Table 3, Fig. 2).

As previously mentioned, sample size was drastically increased in the two recent studies in the UK Biobank [14, 15]. Some differences between both studies have been discussed earlier (i.e., sample size and genome-wide significant threshold). Concerning heart rate recovery, it is worth mentioning that the phenotype definition was not equal between both studies. The study of Ramirez et al. [15] determined heart rate recovery traditionally as the difference between maximum heart rate and heart rate approximately 1 min after cessation of exercise. The study of Verweij et al. defined heart rate recovery at five time points, which included the differences between maximum heart rate and heart rate after 50, 40, 30, 20, and 10 s after exercise. This includes heart rate recovery at earlier time points (i.e., 10 s), which was recently established to be a superior predictor of outcome of all-cause mortality and death by coronary artery disease [6, 7].

Interestingly, both studies found the previously investigated candidate gene *CHRM2* to be associated with heart rate recovery [14, 15]. *CHRM2* encodes M2R, the main muscarinic cholinergic receptor in the heart. This receptor is known for both its negative chronotropic and inotropic effects after binding with acetylcholine released by postganglionic parasympathetic nerves (Table 3, Fig. 2) [68]. The role of the parasympathetic nervous system in interindividual differences in heart rate recovery is additionally highlighted by the *ACHE* gene that was found in both studies. *ACHE* encodes for acetylcholinesterase, an enzyme which breaks down acetylcholine in the synaptic cleft of postganglionic parasympathetic nerves [69]. An increase of acetylcholinesterase would therefore cause an attenuated heart rate recovery by decreasing parasympathetic reactivation. Other genes that were found in both studies were *SYT10, CNTN3, PAX2, CAV2, MED13L, RNF220,* and *NDUFA11* (Table 3, Fig. 2)*. SYT10* encodes a Ca^2+^ sensor synaptotagmin 10 that triggers IGF-1 exocytosis, which, in turn, protects neurons from degeneration. *SYT10* might play an important role in the regulation of heart rate, as it was found to be associated with resting heart rate [3, 23], heart rate increase [15], and heart rate variability [60] as well. *CNTN3* belongs to a group of glycosylphosphatidyl-anchored cell adhesion molecules that are mostly found in neurons [70, 71]. Because of its similarity with *TAG*-*1*, it is thought to have an important function in neuronal outgrowth and wiring of the nervous system [70–72]. In the study of Ramirez et al. it was found that the allele of one SNP decreased heart rate recovery and increased *CNTN3* expression levels in the nucleus accumbens [15]. Since heart rate recovery is mainly influenced by the parasympathetic nervous system [64], it was hypothesized that CNTN3 may also be relevant to cardiac parasympathetic modulation [15]. However, it is more likely to be associated with cardiac sympathetic modulation, since morphology of the nucleus accumbens has been shown to be correlated with cardiac sympathetic index [73]. *PAX2* is known to be the first gene to be expressed in the mid- and hindbrain during embryonal developments in mice [74] and can be found in the hindbrain in the early stages of embryo development in humans as well [48]. The hindbrain includes the nucleus tractus solitarius, nucleus ambiguous, and dorsal nucleus of the vagus, which are known to mainly influence cardiac parasympathetic innervation of the heart through vagus nerve stimulation [75]. Less is known about *CAV2*, which was found to be associated with heart rate response to exercise as well. However, one study pointed out that *CAV2* is necessary for differentiation of dorsal root ganglion cells during the early differentiating programs [51]. The function of *MED13L* is unclear as well, but knockdown in zebrafish caused abnormal neural-crest cell migration [76]. This is supported by clinical characteristics in humans with *MED13L* mutations, which can be characterized by intellectual disabilities, developmental delay, and craniofacial anomalies [77]. *RNF220* functions as an E3 ubiquitin ligase, which determines protein target specificity during posttranslational ubiquitination [53]. A possible link with heart rate recovery originates from the involvement of *RNF220* in the canonical WNT signalling cascade. In a knockdown study, *RNF220* was shown to stabilize β-catenin by interacting with ubiquitin-specific peptidase Usp7 [54]. This stabilizing function is important, because the WNT/β-catenin signalling pathway is involved in embryonic cardiac development [78], the development of cardiac disease [79–81], and in cardiac repair [80]. *NDUFA11* is an accessory subunit of the mitochondrial membrane respiratory chain NADH dehydrogenase complex I. In humans, a splice‐site mutation in this gene is known to cause mitochondrial complex I deficiency. This can cause a wide range of disorders, including encephalocardiomyopathy [82]. Recently, it was shown that downregulation of *NDUFA11* by small interfering RNA reduced ATP production and increased mitochondria reactive oxygen species production in cardiac mitochondria of mice [83]. *NDUFA11* was found to be associated with heart rate variability as well, suggesting that it is an important factor in causing differences between individuals’ heart rate response [60].

Other candidate genes found in one of the GWASs provide a biological hypothesis for their possible causal role in interindividual differences in heart rate recovery as well. These genes include *CCDC141, BCL11A, KCNH8, ALG10B, GNG11, GRIK2,* and *NEGR1. CCDC141* is a gene that plays a central role in neuronal development [44, 45]. In fact, in utero knockdown of *CCDC141* in mice resulted in impaired radial migration in [44]. The same applies to *BCL11A*, which encodes a C2H2-type zinc-finger protein that is involved in neuronal development. Studies in mice have shown that slowed migration of neurons upon knockdown resulted in microcephaly with decreased brain volume [84], particularly affecting the limbic system [85]. Within the human brain, it is most highly expressed in the caudate nucleus followed by hippocampus [86]. In humans, different de novo heterozygous mutations have been found to cause developmental disorder with persistence of fetal haemoglobin [85]. *KCNH8* encodes a voltage-gated potassium channel. It is mainly expressed in the central nervous system and is involved in the regulation of neuronal excitation (Table 3, Fig. 2) [87–89]. *ALG10B* is involved in potassium regulation, as well, since it is a potassium channel regulator that couples to *KCNH2*. However, it is more involved in cardiac tissue than neuronal tissue and is known for its influence on heart rhythm. Upon binding with *KCNH2,* it reduces sensitivity to classic proarrhythmic drug blockade [90]. *GNG11* encodes the γ11 subunit of the heterotrimeric G protein complex Gαβγ [91]. *GNG11* is just as *RGS6* thought to be involved in GIRK activation and was found to be associated with heart rate variability [60] as well. In this study, it was hypothesized that variations in this gene lower the availability of the γ11 subunit, thereby reducing Gαβγ component-induced GIRK activation [60]. This would lead to decreased heart rate variability through attenuated response to changes in cardiac vagal activity [60]. If true, the same would apply for heart rate recovery; decreased response to cardiac vagal reactivation after exercise would translate to blunted heart rate recovery. In addition, another mutation in the *RGS6* gene in humans was shown to decrease susceptibility to the long QT syndrome [92]. *GRIK2* encodes a glutamate receptor that is mostly expressed in the human cerebral and cerebellar cortices [93]. Here, it is involved in neuronal excitation and plays an important role in a variety of normal neurophysiologic processes. Neuronal Growth Regulator 1 (*NEGR1*) is essential for neuronal morphology and, just as *CNTN3*, has been shown to regulate neurite outgrowth (Table 3, Fig. 2) [94]. Perhaps because of this essential function, *NEGR1* has been associated with many polygenetic traits, including body mass index, years of education, and physical activity.

Heart rate increase and recovery share a high genetic correlation and it is therefore likely that there is overlap in genes that were found for both aspects of the heart rate response to exercise [14]. *SNCAIP, SOX5, RGS6,* and *MCTP2* genes were already discussed for heart rate increase during exercise because of their stronger association with this phenotype.

*BCAT1, CLPB, PRDM6, SKAP,* and *SERINC2* are also shown in Table 3, but have not been discussed yet. To our knowledge, these genes could not be linked to heart rate recovery after exercise on a biological basis so far.

## Long-term modification of the heart rate response to exercise

### Heart rate increase

Regular endurance exercise training is known to shift the cardiac autonomic balance towards vagal dominance [10] and, as a consequence, diminish submaximal heart rate when an individual cycles at the same intensity [11]. Large interindividual differences were observed for submaximal heart rate training response [95] and heritability analysis estimated a genetic component ranging between 0.34 and 0.36 (Table 1) [16, 17]. Therefore, several studies were conducted to gain insights in the causes of these interindividual differences. The first study in the HERITAGE family cohort found a heritability of 0.34 for exercise heart rate changes to regular training, with the strongest linkage on chromosome 2q33.3-q34 [17]. Next, this region was fine-mapped and it was found that the *CREB1* gene locus was strongly associated with submaximal exercise heart rate training response [96]. Nonetheless, it only explained 5.45% of the 34% heritability [96].

To gain further insights in the genes causing the remaining fraction of its heritability, a GWAS was performed in the HERITAGE family cohort. In this study, nine SNPs were identified and accounted for the total of 34% heritability of exercise-induced changes to heart rate increase [97]. The most significantly associated SNP was linked to the *YWHAQ* gene (Table 4). *YWHAQ* is mostly expressed in the brain, heart, and pancreas [98], and its main function is apoptosis and cell proliferation. It was shown that the cardiac-specific mutated *YWHAQ* gene leads to increased pathological ventricular remodelling with increased cardiomyocyte apoptosis after experimental myocardial infarction [99]. It can be hypothesized that mutations in the *YWHAQ* gene lead to similar pathological cardiac remodelling after exercise training, causing diminished exercise-induced changes to heart rate increase. However, a neurological causal pathway cannot be ruled out, since the same mechanism could apply to neuronal remodelling needed to attenuate heart rate increase after regular exercise training [11].The *CREB1* gene (Table 4) was significantly associated with submaximal heart rate response to exercise training as well [97]. In this study, it was hypothesized that *CREB1* altered the exercise-induced changes in heart rate increase due to its effect on either cardiac [100] or neuronal memory [101]. Cardiac memory is a phenomenon in which an altered T wave on electrocardiogram can be seen when sinus rhythm restarts after a period of abnormal rhythm, for example, after ventricular pacing or arrhythmia [100]. The other hypothesis involving neuronal memory fits in our current understanding that neuron biology is of great importance in the heart rate response to exercise. Neuronal memory or long-term potentiation is a form of synaptic plasticity in which there is a long-lasting increase of synaptic strength in case the synapse is highly active. It could be hypothesized that regular exercise causes an increase of synaptic strength of parasympathetic neurons, thus altering the heart rate increase during exercise. However, *CREB1* encodes a transcription factor that regulates many mechanisms in the body and its association with memory does not imply causality. A recent editorial rightfully addressed the fact that the same allele in another study was found to increase the rise of temperature [102] and, therefore, might decrease subjective liking of exercise training, potentially diminishing motivation [103].

**Table 4:** Summary of genes involved in the long-term heart response to exercise

Gene	Variant	Chromosome/position	*P* value	Type of study	Training schedule	Type of exercise test	Population	Author; year
Heart rate increase								
CREB1^a^	rs2253206	2:208100223	1.6 × 10^−5^	GWAS	20 weeks, 3 times a day, 30–50 min at submaximal HR	Submaximal bicycle	Healthy, but sedentary	Rankinen et al. (2012) [97]
GCH1^a^	rs2057368	14:54373759	5.6 × 10^−5^	GWAS	20 weeks, 3 times a day, 30–50 min at submaximal HR	Submaximal bicycle	Healthy, but sedentary	Rankinen et al. (2012) [97]
GPRIN3^a^	rs1560488	4:90444858	3.3 × 10^−5^	GWAS	20 weeks, 3 times a day, 30–50 min at submaximal HR	Submaximal bicycle	Healthy, but sedentary	Rankinen et al. (2012) [97]
RBPMS^a^	rs2979481	8:30382328	3.8 × 10^−6^	GWAS	20 weeks, 3 times a day, 30–50 min at submaximal HR	Submaximal bicycle	Healthy, but sedentary	Rankinen et al. (2012) [97]
MYLIP^a^	rs909562	6:16238312	3.2 × 10^−5^	GWAS	20 weeks, 3 times a day, 30–50 min at submaximal HR	Submaximal bicycle	Healthy, but sedentary	Rankinen et al. (2012) [97]
OR6N2^a^	rs857838	1:157017174	7.6 × 10^−5^	GWAS	20 weeks, 3 times a day, 30–50 min at submaximal HR	Submaximal bicycle	Healthy, but sedentary	Rankinen et al. (2012) [97]
PIWIL1^a^	rs4759659	12:129403241	5.7 × 10^−5^	GWAS	20 weeks, 3 times a day, 30–50 min at submaximal HR	Submaximal bicycle	Healthy, but sedentary	Rankinen et al. (2012) [97]
TFEC^a^	rs10248479	7:115395591	3.4 × 10^−5^	GWAS	20 weeks, 3 times a day, 30- 50 min at submaximal HR	Submaximal bicycle	Healthy, but sedentary	Rankinen et al. (2012) [97]
YWHAQ^a^	rs6432018	2:9639347	8.1 × 10^−7^	GWAS	20 weeks, 3 times a day, 30–50 min at submaximal HR	Submaximal bicycle	Healthy, but sedentary	Rankinen et al. (2012) [97]
Heart rate recovery								
CHRM2^b^	rs324640	7:136146251	0.008	Candidate	2 weeks, 5 times a week, 40 min at submaximal HR	Maximal bicycle	Healthy, but sedentary	Hautala (2006) [66]
CHRM2^b^	rs8191992	7:136158563	0.005	Candidate	2 weeks, 5 times a week, 40 min at submaximal HR	Maximal bicycle	Healthy, but sedentary	Hautala (2006) [66]

Genes found to be associated with changes in training-induced changes to heart rate increase and recovery are shown in alphabetical order. Variant stands for either a SNP or deletion/insertion mutation. Candidate stands for candidate gene study. GWAS stands for genome-wide association study

^a^Allele frequencies and betas are not mentioned in this study and direction (in- or decrease of response to training) can, therefore, not be determined

^b^Minor alleles of rs324640 and rs8191992 (respectively, A and C) decreased heart rate recovery

### Heart rate recovery

On the other hand, heart rate recovery increases when the cardiac autonomic balance shifts towards vagal dominance after regular endurance training [10]. Little research has been performed on the genetics of training-induced changes to heart rate recovery, although a heritable component has been suggested [66]. To our knowledge, only one study has been conducted on this subject. In this candidate gene study, it was found that the *CHRM2* gene (Table 4) is linked to long-term modification of heart rate recovery to exercise training as well [66]. Participants who had a the minor alleles of the rs324640 and rs8191992 SNPs were not only found to have a lower acute heart rate recovery, but also showed less increase in heart rate recovery after regular endurance training. As previously mentioned, *CHRM2* encodes the muscarinic acetylcholine receptor M2R and, upon activation, causes a negative chronotropic and inotropic response. It therefore seems that genetic variation in *CHRM2* not only causes interindividual differences in acute heart rate recovery [68], but also in long-term modifications. A full overview of the genes discussed for the long-term heart rate response to exercise can be found in Table 4.

## Association of heart rate response to exercise-related genes with other traits

We assessed the association of described genes with other traits in publicly available GWASs using the GWAS catalogue (Online Resource 1). In short, the candidate causal genes that were associated with both heart rate increase and recovery were also associated with resting heart rate (*CCDC141*, *RGS6*, *RNF220*, *SCN10A*, and *SYT10*), heart rate variability (*CCDC141*, *RGS6*, *RNF220*, and *SYT10*), blood pressure (*CCDC141* and *PAX2*), atrial fibrillation (*CAV2* and *SCN10A*), coronary artery disease (*CAV2* and *SCN10A*), and ECG traits including the PR interval (*CAV2* and *SCN10A*), QRS duration, and the Brugada syndrome (both *SN10A*).

Some genes that were only associated with heart rate increase during exercise were found to be associated with resting heart rate and heart rate variability (*PPIL1*), blood pressure (*ADRB1*, *ACE*, *NOS3*, and *HMGA2*), atrial fibrillation (*MCTP2* and *NOS3*), exercise treadmill test and lung function (both *RYR2*). Similarly, some of the heart rate recovery genes were also associated with resting heart rate (*ACHE* and *GNG11*), heart rate variability (*GNG11* and *NDUFA11*), blood pressure (*PRDM6*, *PRKAG2*, and *CHRM2*), QRS duration, (*PRDM6*), atrial fibrillation and coronary artery disease (*BCL11A*, *PRDM6*), and obesity and vigorous physical activity levels (both *NEGR1*).

## Future directions

**Fig. 3:**
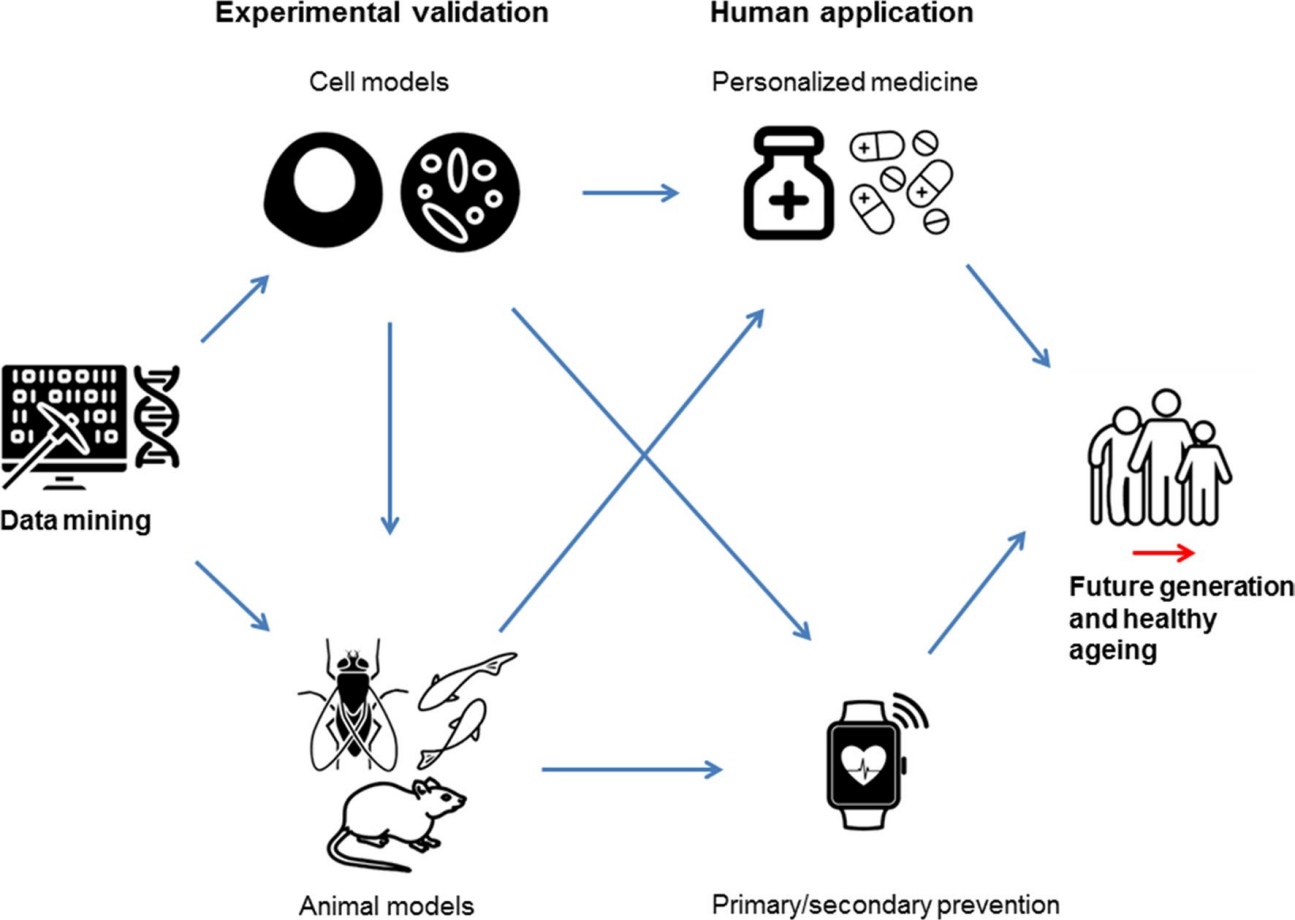
Possible follow-up of GWAS on heart rate response to exercise. Cell models based on pluripotent stem cells provide a potential functional model to study GWAS findings using experimental manipulations that cannot be performed in vivo. Complex mechanisms of genetic interplay could be studied in animals that share a high percentage of their genomic sequence with humans, including mice, fruit flies, and zebrafishes. Tools such as gene knockdowns can be used to manipulate the genomes of these animal models. The ultimate application of knowledge initiated by GWAS findings in heart rate response to exercise lies in the improvement of primary and secondary prevention and personalized medicine to improve human health

Improvement of prevention and treatment of disease in the human health sector is the ultimate application of novel knowledge found by genetic studies and future research should be performed to achieve this goal **(**Fig. 3) [104]. Functional follow-up of findings obtained by GWAS will be necessary to gain insights in how likely causal genes affect the heart rate response to exercise [104]. Most genes that were prioritized so far have a plausible biological mechanism in which they influence the heart rate response to exercise. However, the exact effect of all genes on exercise-induced heart rate changes could be validated in an experimental setting (Fig. 3). One possible method is to perform functional experiments in cardiomyocytes obtained from embryonic stem cells [105]. In cardiomyocytes, human diseases and risk factors with their underlying genetic contribution can be created in vitro [105]. Since cardiomyocyte cell cultures can beat spontaneously [105], simulating the effect of this genetic contribution allows for investigation of the acute heart rate response to pacing from resting to exercise heart rate levels in small cell cultures. In addition, by simulating the effect of this genetic contribution, drugs can be screened against an individual’s full genetic backgrounds to discover information on cardiotoxicity for each individual. This could potentially give insights in the development of personalized medicine strategies for heart rate modification [106], which is an essential strategy in the treatment of coronary artery disease [107] and heart failure (Fig. 3) [108, 109]. Genes known to affect cardiac de- and repolarisation (*RYR2*, *ALG10B,* and *SCN10A*) or GIRK channels in the cardiac sinus node (*RGS6* and *GNG11*) could be of interest to study in this setting. Recent development in the generation of spinal human cord neural cells could provide the same opportunity for investigating neuronal cell longevity including genes such as *SCNAIP*, *POP4,* and *SYT10* [110]. Complex neurological mechanisms at the interplay of the sympathetic and parasympathetic nervous system (i.e., *KCNH8* and *GRIK2*) or neuronal development (i.e., *SOX5*, *PAX2,* and *BCL11A*) are more difficult to investigate using this method. This can be solved by investigating these genes using in vivo models of animals that share a high percentage of their genomic pattern with humans, including mice [111, 112], fruit flies [113], and zebrafish [114] (Fig. 3). For example, knockdown of *RGS6* [56, 57]*, MED13L* [76], and *BCL11A* [85] has already provided insights in biological consequences of mutations in these genes.

Improvement of prevention of disease is another goal of genetic research. While accurate risk prediction might be relatively straightforward for mono- and oligogenic disease, this is more difficult for polygenic diseases such as coronary artery disease and heart failure. However, the knowledge on genetic variants obtained by GWAS can be used to construct genetic risk cores by summing the number of risk alleles weighted by the corresponding beta coefficients. Recently, it was shown that the polygenic risk score of coronary artery disease had the ability to identify 8.0% of the population at greater than threefold risk for coronary artery disease [115]. These individuals can subsequently be selected for encouragement of behavioural lifestyle changes as relative effects of poor lifestyle were shown to be comparable between genetic risk groups [116]. Similar to the traditional risk score models in which several traditional risk phenotypes are used to predict risk events, this could ultimately be performed for genetic risk score models as well. As previously stated, there is a large body of observational studies that links heart rate response to exercise to all-cause mortality and cardiovascular disease in healthy individuals and those with a history of cardiac disease [5–9]. In this light, it would be interesting to see whether adding the polygenetic risk scores for the acute heart rate response to exercise into a genetic risk score model that includes the polygenetic risk score for the cardiovascular disease itself could improve detection of individuals at high risk of disease. However, it should be noted that both recent GWAS on the acute heart rate response to exercise did not find support for a genetic association with cardiovascular mortality [14, 15]. The lack of an association in both studies might originate from the fact that a small replication cohort consisting of a relatively young and healthy population was used. The study of Verweij et al. [14] did find a significant association between heart rate response to exercise and parental age as proxy for all-cause mortality. However, first, it is required to investigate whether there is a genetic association with cardiovascular disease and all-cause mortality, preferably in a larger independent cohort [117].

The evidence on long-term modification of the heart rate response to exercise is limited so far [97]. If the genetics of the acute heart rate response to exercise can be used to predict cardiovascular mortality, the combination with information on the genetics of the long-term modification of the heart rate response to exercise could one day inform the choice of prevention strategy. For example, a high genetic risk score for a diminished acute response to exercise combined with a genetic risk score that indicates high training-induced changes to heart rate response could be an indicator of early primary or secondary prevention strategies (Fig. 3). On the other hand, a high genetic risk score for a diminished acute response to exercise combined with a genetic risk score that indicates little training-induced changes could be an indication of early intervention through medication (Fig. 3).

## Conclusion

In the current review, we found a total of 10 genes associated with the acute heart rate response to exercise in candidate gene studies. Only one gene (*CHRM2*), related to heart rate recovery, was replicated in recent GWASs. Additional 17 candidate causal genes were identified for heart rate increase and 26 for heart rate recovery in these GWASs. Nine of these genes were associated with both acute heart rate increase and recovery during exercise. These genes can be broadly categorized into four categories: (1) development of the nervous system (*CCDC141*, *PAX2*, *SOX5,* and *CAV2*); (2) prolongation of neuronal life span (*SYT10*); (3) cardiac development (*RNF220* and *MCTP2*), and (4) cardiac rhythm (*SCN10A* and *RGS6*). Of the total of 43 genes, nine showed overlap with resting heart rate and heart rate variability, six with atrial fibrillation and coronary artery disease, two with ECG traits, and nine with blood pressure. The current findings support the idea that the autonomic nervous system is a major player in the regulation of the acute heart rate response to exercise. Heart rate recovery is especially influenced by parasympathetic nervous system genes (*ACHE* and *CHRM2*), in line with the previous research [64]. Regarding the long-term response to exercise, heart rate increase during exercise was found to be mainly associated with genes involved in either cardiac or neuronal remodelling. Little evidence has been found for the long-term response of heart rate recovery to exercise, except for parasympathetic involvement. Future work will be required to translate these findings to preventive and therapeutic applications.

### Electronic supplementary material

Below is the link to the electronic supplementary material.

Supplementary material 1 (XLSX 143 kb)
